# Editorial: Sleep disorders in neuromuscular diseases: treatable conditions: the evolving scenario of sleep in neuromuscular disorders

**DOI:** 10.3389/fneur.2024.1448486

**Published:** 2024-07-11

**Authors:** Corrado Italo Angelini, Gabriele Siciliano, Carl Ansevin

**Affiliations:** ^1^University of Padua, Padua, Italy; ^2^University of Pisa, Pisa, Tuscany, Italy; ^3^Independent Researcher, Youngstown, OH, United States; ^4^Ohio Neurologic Institute, Youngstown, OH, United States

**Keywords:** sleep, SMA, charcot-marie tooth, advances, drug, sleep apnea, rapid-eye movement REM

Modifications of sleep have been an active field of investigation in severe neuromuscular disorders (NMD). The relevance of these conditions needs evaluation since they might contribute to sleep-disordered breathing (SDB). Sleep plays a crucial role in the overall health and wellbeing of individuals, including people with NMD.

Neuromuscular disorders affect motor neurons, the nerves that control voluntary muscles, leading to symptoms characterized by muscle weakness, and fatigue. The pathophysiology is that muscle repair and recovery occur during sleep, and the body undergoes processes related to muscle repair. Adequate sleep allows the muscles to repair and regenerate, which is essential for people with NMD who may experience muscle degeneration or weakness.

Sleep is a period of reduced energy expenditure, allowing the body to conserve energy for essential functions. NMD affects energy levels and muscle function, sufficient sleep can help optimize energy resources. Pain management during sleep plays a role in pain modulation and perception. Quality of sleep is important for managing pain association syndromes such as neuropathy.

There is an evolving scenario because of new therapy progress in several NMDs, such as spinal muscular atrophy (SMA).

Sleep disorders have been an active field of investigation in a collection of papers, in Research Topic: “*Sleep disorders in neuromuscular diseases: treatable conditions*” There were several outstanding contributions:

Abati et al. have reviewed sleep patterns in SMA. While assisted ventilation has improved survival, it might result in ventilator dependence. The development of new SMN-augmenting therapies has renewed optimism, but their long-term impact on respiratory function is still uncertain, and non-invasive respiratory support remains part of SMA management. Despite the importance of respiratory support in SMA, knowledge regarding sleep disorders in this population is limited. This contribution covers sleep-related breathing disorders in patients with SMA, with a focus on SMA type 1 and progress in treatment. Major advances have changed the perspective for both neonatal SMA and children, demanding neonatal screening. Novel drugs are increasingly used to target specific molecules involved in these disorders. For example, nusinersen (1) and risdiplam have been developed to target RNA splicing defects in SMA (2).

There are three approved treatments for spinal muscular atrophy: these are Nusinersen (Spinraza), an antisense oligonucleotide (ANO), Onasemnogene abeparvovec-xioi (Zolgensma), an SMN1 gene replacement, and Risdiplam (Evrysdi), a small molecule SMN2 modifier. The modification in SMA with such therapies was part of this evolving topic.

To investigate the role of sleep in Charcot-Marie-Tooth (CMT) disease Massucco et al. reported a case report of twins with CMT due to PMP22 duplication, with sleep-disordered breathing. This case report related to sleep disorders suggested that it may be an under-recognized feature in CMT. Sleep apnea may be an additional feature of CMT that we should look into. The twins harbored PMP22 duplication with typically demyelinating features in the median and peroneal motor nerves. The Apnea Hypopnea Index and Body Mass Index in both patients were mildly elevated. The patients exhibited signs of nocturnal hypoventilation and phrenic nerve damage. Several factors, including sleep apnea, may contribute to SDB in patients with CMT. A thorough evaluation may differentiate other causes of SDB and suggests that patients with CMT may be predisposed to developing sleep apnea.

Hoxhaj et al. did a comprehensive analysis of excessive daytime sleepiness (EDS) in myotonic dystrophy type 1 (DM1) and type 2 (DM2). EDS is a common and debilitating symptom in both forms of myotonic dystrophy, significantly impacting patients' quality of life. Polysomnographic studies have revealed a prominent dysregulation of REM sleep in DM1, suggesting a possible narcoleptic-like phenotype. Additionally, alterations in NREM sleep, including increased sleep instability and impaired delta power dissipation contribute to daytime sleepiness. Besides motor outcome measures, a group of self-reported outcome measures is described by Simoncini et al. (3). Some were developed for use in DM1 patients, including DM1- ActivC, a measure of capacity for activity and social participation, and the fatigue and daytime sleepiness scale. While SDB and respiratory dysfunctions are prevalent in DM1 and DM2, their direct correlation with EDS remains complex and inconclusive. Imminent are trials with AON in DM1 since several AONs achieved a satisfactory safety profile and dose-dependent skeletal muscle delivery. In planned trials, the key endpoint is video measurement of grip strength while other secondary endpoints are QMT total score, and activities of daily living as measured by DM1-Activ, QoL.

The role of sleep was part of a minireview that indicated when polysomnography might be required to evaluate the reduction in REM sleep, and loss of normal atonia during REM in individual DM1 patients, which in single cases might have a paradoxical beneficial effect and protect against SDB. On this basis, several risk factors appear with increasing age in several NMDs and differ where sleep is a normal restorative function. Untreated sleep disorders in NMD patients can lead to a deterioration in their condition over time.

The relevance of these latter considerations needs further evaluation. SDB can occur covertly with conditions like fibromyalgia, metabolic syndromes, or neurogenic respiratory conditions like ALS. Sleep disorders may arise in NMDs including insomnia due to pain or leg muscle cramps, restless legs syndrome, obstructive sleep apnea, and hypoventilation.

## Author contributions

CIA: Conceptualization, Data curation, Writing – original draft. GS: Data curation, Writing – review & editing. CA: Conceptualization, Data curation, Writing – review & editing.
